# An Oscillatory Neural Autoencoder Based on Frequency Modulation and Multiplexing

**DOI:** 10.3389/fncom.2018.00052

**Published:** 2018-07-10

**Authors:** Karthik Soman, Vignesh Muralidharan, V. Srinivasa Chakravarthy

**Affiliations:** ^1^Bhupat and Jyoti Mehta School of Biosciences, Department of Biotechnology, Indian Institute of Technology Madras, Chennai, India; ^2^Department of Psychology, University of California, San Diego, San Diego, CA, United States

**Keywords:** oscillatory autoencoder, Kuramoto oscillator, adaptive Hopf oscillator, frequency modulation, multiplexing, phase synchronization, EEG

## Abstract

Oscillatory phenomena are ubiquitous in the brain. Although there are oscillator-based models of brain dynamics, their universal computational properties have not been explored much unlike in the case of rate-coded and spiking neuron network models. Use of oscillator-based models is often limited to special phenomena like locomotor rhythms and oscillatory attractor-based memories. If neuronal ensembles are taken to be the basic functional units of brain dynamics, it is desirable to develop oscillator-based models that can explain a wide variety of neural phenomena. Autoencoders are a special type of feed forward networks that have been used for construction of large-scale deep networks. Although autoencoders based on rate-coded and spiking neuron networks have been proposed, there are no autoencoders based on oscillators. We propose here an oscillatory neural network model that performs the function of an autoencoder. The model is a hybrid of rate-coded neurons and neural oscillators. Input signals modulate the frequency of the neural encoder oscillators. These signals are then multiplexed using a network of rate-code neurons that has afferent Hebbian and lateral anti-Hebbian connectivity, termed as Lateral Anti Hebbian Network (LAHN). Finally the LAHN output is de-multiplexed using an output neural layer which is a combination of adaptive Hopf and Kuramoto oscillators for the signal reconstruction. The Kuramoto-Hopf combination performing demodulation is a novel way of describing a neural phase-locked loop. The proposed model is tested using both synthetic signals and real world EEG signals. The proposed model arises out of the general motivation to construct biologically inspired, oscillatory versions of some of the standard neural network models, and presents itself as an autoencoder network based on oscillatory neurons applicable to time series signals. As a demonstration, the model is applied to compression of EEG signals.

## Introduction

Despite decades of research, the question of neural code is still controversial. Currently there are two well-accepted approaches to the problem: the spike rate code and the spike timing code. The former assumes that the neural code lies in the spike rate and has given rise to large class of rate-coded neural networks (Lippmann, 1989; Ruck et al., 1990; Lawrence et al., 1997). The latter holds that the code lies in the spike timing and has led to creation of a large class of spiking neuron networks (Maass, 1997b; Izhikevich, 2003, 2004; Ghosh-Dastidar and Adeli, 2009). Both rate-coded and spiking neuron networks are endowed with universal computational properties (Maass, 1997a; Auer et al., 2008). However the basic functional unit of the brain seems to be, not a single neuron, but a “cell assembly” (Buzsáki et al., 2012), a cortical column being an example of such a unit (Buzsáki and Draguhn, 2004). The collective activity of a cell assembly is not a spike train but a smoother signal called the local field potential (LFP) (Buzsáki et al., 2012). Most of the functional neuro–imaging data including the electroencephalogram (EEG) and functional Magnetic Resonance Imaging (fMRI) encompass the description of the neural activity at this level (Logothetis et al., 2001; David and Friston, 2003; Whittingstall and Logothetis, 2009). Thus when it comes to the description of neural activity at the level of cell assemblies the standard tools and concepts of signal processing could be deployed. The activity of a single cell assembly can then be described in terms of amplitude, frequency, and phase. Communication between two cell assemblies can be described in terms of phase difference at a given frequency. Hence observed neuro physiological phenomena may be explained in terms of oscillator entrainment and phase synchronization.

It is then natural to envisage neural models of three broad classes—rate code based, spike-based, and oscillator based. There are indeed neural models of oscillators (Wang and Terman, 1995; Campbell et al., 1999; Ijspeert, 2008) but they seem to be often applied to specialized purposes and do not seem to enjoy the universality of both rate coded and spiking neuron network models. Oscillatory neuron models are used to model extensively oscillatory phenomena of the brain like building generative models of cortical oscillations to understand brain rhythms and neuronal synchronization (Cumin and Unsworth, 2007; Breakspear et al., 2010). Furthermore when it comes to modeling behavior, they are also restricted to those behaviors that are intrinsically rhythmic like the locomotor movements, rhythmic hand movements, or swimming movements (Ijspeert et al., 2005; Ijspeert, 2008). Such restricted use of oscillator models is untenable since the very same brain oscillations which drive the hand when making rhythmic tapping movements also enable it to perform non-rhythmic point-to-point reaching movements. Although there are exceptions to this case (see Hoppensteadt and Izhikevich, 2000; Heitmann et al., 2015) there exist only a minimal literature on using oscillatory dynamics to explain non-oscillatory behavior. Therefore it is important to investigate if oscillatory neural network models possess the property of universal computation that forms the core strength of its rival models: rate-coded and spiking neural network models.

The strength of the rate coded and spiking neuron networks lies in the fact that they have been designed to solve a wide of range of useful information processing problems: to construct transformations from one space to another (Lippmann, 1989; Schmidhuber, 2015), to map high dimensional information onto bounded two-dimensional spaces (Kohonen, 1998), to process sequences (Frasconi et al., 1995), to store patterns as attractors (Hopfield, 1982; Trappenberg, 2003), to construct dimensionality reduced representations by autoencoding (Oja, 1989; Sanger, 1989; Hinton and Salakhutdinov, 2006) and so on. In this realm of applications, in most cases, equivalent oscillatory neural network models have not been designed which, when realized, could form another dimension for understanding standard neural network theory.

Apart from the aforementioned research on neural codes, in the realm of neural signal processing, it becomes natural to link the brain signals arising from EEG and MEG to an underlying oscillatory process which connects to the mechanistic underpinnings of brain circuitry. Utilizing these ideas, a large body of literature exists in the domain of EEG related applications like Brain Computed Interfaces (BCIs). Often in these studies motor imagery EEG signals are recorded, classified and the results of classification are used to drive a machine like the wheelchair (Leeb et al., 2007a,b). The dependence on the stationarity of signals is very important for current methods, including optimal spatial filtering (Ramoser et al., 2000) to solve these class of problems posing difficulty in reliable processing of EEG. The stochastic and non-linear nature of EEG signal thus poses critical challenges in its processing such as feature extraction and further classification (Pfurtscheller and Neuper, 2001). As of now, there exists no benchmark method to decipher this problem of EEG processing. We believe that a better understanding of the oscillatory neural network models, mimicking the underlying neural process, could pave way to a novel class of algorithms for processing EEG signals.

Although the objective of the proposed model is to shed light on the oscillatory neural code, we would also like to briefly cite literature on time series data mining and time series representations. Time series data mining is apparently a challenging one because of the unique characteristic features of the time series data such as presence of noise, and non-linear relation of the data elements (Wilson, 2017). A problem that often arises in time series data processing is to form an optimal representation of the data either by reducing or approximating it, but making sure that the approximated version of the data still carries the local/global features of the original version. For the ease and efficient use of the data, the main challenge is to choose an optimal representation of the same. Time series data representation is a well-studied area where methods such as Discrete Fourier Transform (DFT) (Faloutsos et al., 1994), Discrete Wavelet Transform (DWT) (Percival and Walden, 2006), time series Piecewise approximation (Keogh et al., 2001a,b) have been proposed. Due to the current trends in the use of “big data” processing, other novel methods such as transformation of the time series data to discrete variables or symbols has become popular (Lin et al., 2007). The main idea behind this type of methodology is to transform time series data to a sequential data of symbols by initially discretizing the time series using methods like Piecewise Aggregate Approximation (PAA) (Keogh et al., 2001b). This can be treated as a way to reduce the number of points in the time series data and this is followed by converting the approximated numerical data to corresponding symbols using popular algorithms like SAX (Lin et al., 2007). The advantage of converting the time series to symbolic sequences is that, once the transformation is made, standard pattern matching algorithms can be applied to the sequences for further processing. The aforementioned methods are successful in data mining area, but carry little information on the neural processing of time series data. This is not a flaw of the aforementioned methods because they are not intended to provide any neural perspective on time series data processing. However, the real brain is adept at time series processing since most of the sensory inputs coming from different sensory modalities such as vision, proprioception, auditory, vestibular, tactile, and olfactory stimulus are dynamic in nature. Hence, the objective of this study is to propose a computational model that implements the autoencoding of time series data using biologically plausible neural principles. The very next sub section named as “background” gives the impetus behind the proposed modeling architecture.

### Background

In response to the aforementioned general motivation, we now present a network of neural oscillators that serves as an oscillatory autoencoder. The reason why we choose the autoencoder architecture is due to the function it serves i.e., encoding the high dimensional input to a low dimensional abstract representation and further decoding it back to the original input signal. From a neural perspective this can be broadly viewed as different stages of neural information transfer. The first stage starts with the encoding of high dimensional sensory stimulus coming from multiple sensory modalities to a more compatible abstract representation in the subcortical structures. For example, visual information fetched by ~125 million retinal photoreceptors converge to ~1 million neurons of the lateral geniculate nucleus in the thalamus (Hubel, 1995). This is one of the instances (among many) of huge dimensionality reduction that takes place in the real brain. The decoder can be viewed as the stage in which the information is transferred from the subcortical structures to other cortical structures with more number neurons i.e., transfer of information from lower dimension to higher dimension (Guillery and Sherman, 2002). Standard autoencoder networks use static neurons that have limitations in capturing the temporal features of the input in a naturalistic fashion. The proposed model uses the dynamics of oscillatory system such as phase synchronization, frequency tuning, and also uses the signal processing concepts such as frequency modulation (FM) and multiplexing (MUX) to shed light on the possible information transfer mechanisms in the brain.

We brief out here the methods that are adopted to accomplish the aforementioned objective (this is explained in detail in the following methods section). In this model, a set of band-limited signals are frequency modulated by a layer of neural oscillators, multiplexed by a layer of rate-coded neurons, and subsequently demultiplexed and demodulated by oscillatory neurons. The network is a hybrid model consisting of two kinds of oscillator models (Kuramoto and Hopf oscillators) and rate-coded dynamic neurons. The signals obtained at the output of the MUX stage may be considered as a reduced-dimensional representation of the input signals. Finally we test the model on actual EEG signals (real world data). The paper is outlined as follows. Section II presents the methods and the model equations, followed by the results in Section III and finally the discussion in Section IV.

## Methods

**Figure 1:**
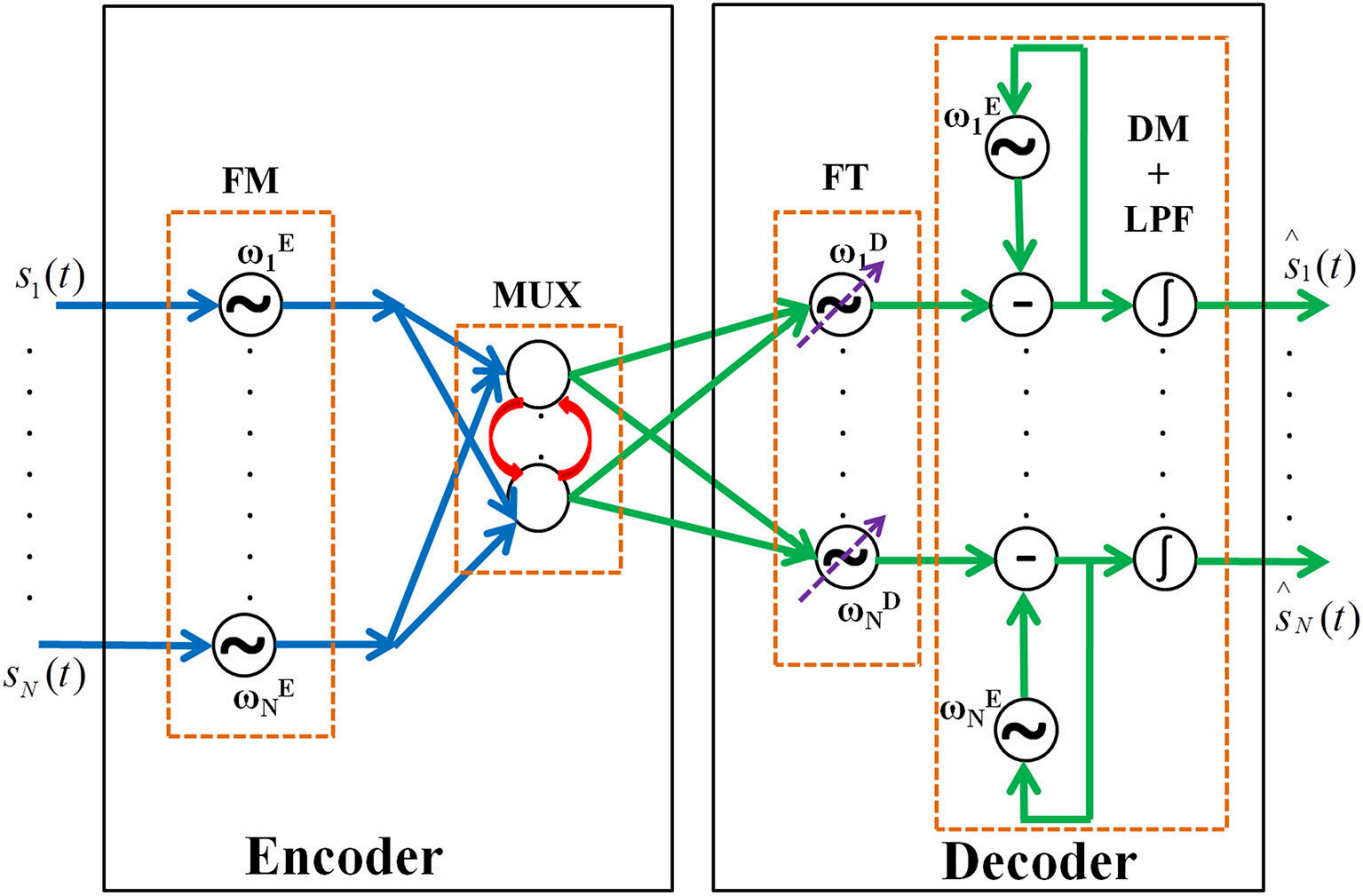
The network architecture of the oscillatory autoencoder network. In the encoder module, the incoming message signals (*s*_1_,…, *s*_N_(*t*)) are encoded, via FM, onto carrier signals with intrinsic frequencies $\omega_{1}^{\text{E}}$,…, $\omega_{\text{N}}^{\text{E}}$. A lateral anti-Hebbian layer is used for frequency multiplexing (MUX) of the modulated signals. In the decoder module, frequency tracking (FT) is performed by a series of adaptive Hopf oscillators which can tune their intrinsic frequency dynamically, followed by a layer of Kuramoto oscillators which synchronize to the frequency of the Hopf oscillators to form a basic unit which extracts the embedded input signals (DM). This is further passed to a leaky integrator (LPF) to get smoothened output of the network [ŝ_1_,…, ŝ_N_(*t*)].

Here we propose the architecture of an autoencoder using oscillatory neurons. The motivation for an oscillatory autoencoder is explained above in the introduction section. The model architecture described here consists of Encoder and Decoder modules as shown in Figure 1. The encoder process the input signals and makes a lower dimensional representation of the same. The decoder module reconstructs back the original input signal from this abstract representation.

The encoder receives inputs as an array of *N* band limited signals, *s*_1_(*t*),., *s*_N_(*t*). These signals are frequency modulated and multiplexed by the encoder. The multiplexed signals are demultiplexed and demodulated by the decoder. Both the encoder and the decoder are networks of oscillators. The networks are hybrids of Hopf and Kuramoto oscillators (Kuramoto, 1984; Righetti et al., 2006). The motivation for choosing two different phase oscillators is described in the decoder section. The encoder and decoder modules are modeled as follows.

### A. encoder

The encoder has two stages viz. Frequency Modulation (FM) stage and MUX stage.

### FM stage

FM stage has *N* phase oscillators each with different intrinsic frequencies. *N* is equal to the dimension of the input. Each of the input signals is connected to one of these oscillators. Input is encoded by the phase dynamics as given in (1). This phase dynamics is equivalent to FM (Haykin et al., 1989) and hence the name FM stage.

(1) $$\begin{array}{l} \overset{•}{\theta_{i}}=\omega_{i}^{E}+s_{i}\left(t\right) \end{array}$$

θ_i_ is the phase of the *i*th oscillator in the encoder layer. $\omega_{\text{i}}^{\text{E}}$ is the intrinsic angular frequency of the *i*th oscillator in the encoder layer. (Note: The superscript E stands for Encoder layer).

### MUX stage

A classical MUX in electronics literature ensures harmonious transfer of information between the sender and receiver by acting like a multiple switch (Omotayo, 1985). Hence, a MUX usually has *n* number of input lines and 1 output line. However, in the proposed model we do not use this strict definition of MUX instead we take the idea of compressing the n input signals to m dimensions where *m* < *n*. This is what is exactly achieved through the hidden layers of a traditional autoencoder. The reason why we named it MUX is to bring about a direct comparison of neural information transfer to the radio FM communication principles.

The MUX stage is implemented by a neural network architecture known as Lateral Anti-Hebbian Network (LAHN) that has Hebbian (excitatory) afferent and anti-Hebbian (inhibitory) lateral connections (Földiak, 1990). The dynamics of a neuron in LAHN is given by Equation (2). Hebbian learning applied to the afferent weight connections (Equation 5) brings the afferent weight vector close to the input data ensuring feature selection by that particular neuron. The anti-Hebbian rule applied to the lateral connections induces competition among the LAHN neurons. Hence each LAHN neuron learns different features from the input data. This network was shown to extract optimal features from the input data by converging transformation weight vectors to the subspace of the principal components of the input data (Földiak, 1990). Since this network maximizes the variance of the output (Földiak, 1990), it extracts optimal features from the input data. The information required for the unsupervised learning of LAHN neuron is available locally at its synaptic connections (Equations 4, 5) and this makes the network biologically plausible.

This LAHN layer acts as the hidden layer for the oscillatory autoencoder. The low-dimensional representations constructed by the hidden layer of a traditional autoencoder are constructed by this MUX stage in the proposed model. Hence, the number of inputs going to the MUX layer is same as the number of encoder oscillators in the *FM stage* and the number of outputs from the MUX should be essentially lesser in number than its input to achieve a dimensionality reduction.

The dynamics of a neuron in the MUX stage is given in (2) and (3).

(2) $$\begin{array}{l} Y_{i}\left(t\right)=\overset{N}{\underset{j=1}{\sum }}q_{ij}O_{j}\left(t\right)+\overset{n}{\underset{k=1}{\sum }}w_{ik}Y_{k}\left(t-1\right) \end{array}$$

(3) $$\begin{array}{l} O_{j}=\sin\left(\theta_{j}\right) \end{array}$$

*Y*_i_ is the output of *i*th neuron, *q* and *w* are the afferent and lateral weight connections of MUX respectively, *N* is the dimension of the input, *n* is the total number of neurons in the LAHN. *O*_j_ is the state of the *j*th input oscillator. In MUX, lateral weights are updated using anti-Hebbian learning and afferent weights are updated using Hebbian learning (Földiak, 1990) as given in (4) and (5).

(4) $$\begin{array}{l} \Delta w_{ik}=-\eta_{L}Y_{i}\left(t\right)Y_{k}\left(t-1\right) \end{array}$$

(5) $$\begin{array}{l} \Delta q_{ij}=\eta_{F}\left[O_{j}\left(t\right)Y_{i}\left(t\right)-q_{ij}{Y_{i}}^{2}\left(t\right)\right] \end{array}$$

η_L_ and η_F_ are the learning rates for lateral and feed forward weights respectively. MUX with *n* nodes trained using (4) and (5) mixes the input FM signals with a minimal overlap in their frequency spectrums which further decreases the reconstruction error.

### B. decoder

The decoder has three stages such as Frequency Tracking (***FT***) performed by adaptive Hopf oscillators, Demodulation (***DM***) using Kuramoto oscillators, and final smoothening of signal by low-pass filtering (***LPF***) using leaky integrator neurons stages respectively. Each section is explained in detail below.

### FT stage

Initially the responses of MUX are passed onwards to the FT stage. The purpose of this stage is to tease out the individual frequencies which are mixed by the MUX stage. This frequency tracking is achieved by using Hopf oscillators with adaptive frequency dynamics. Hopf oscillators were successfully implemented as an adaptive frequency system that updates its intrinsic frequency in an iterative way until it converges to one of the frequencies of the input data (Righetti et al., 2006). This system of Hopf oscillators was previously shown by Righetti et al. (2006) to learn the frequency components of its input signals. This was achieved by adding a frequency adaptation variable to the classical two variable Hopf oscillator dynamics (Righetti et al., 2006). This was shown in phase oscillators having unit circle phase plane limit cycles i.e., using Hopf oscillators. They have further explained similar frequency adaptation dynamics for relaxation oscillators too. However, in this model we are using harmonic phase oscillators for the frequency tracking stage as explained below.

Here, we wanted to achieve the aforementioned phenomena of tracking the frequency of input data. The adaptive frequency Hopf oscillators act like band-pass filters and filter out different frequency bands from the mixed input signal. The adaptive frequency dynamics is accomplished using the following equations:

(6) $$\begin{array}{l} \overset{•}{r_{i}}=r_{i}\left(\mu -{r_{i}}^{2}\right) \end{array}$$

(7) $$\begin{array}{l} {\overset{•}{\phi }}_{i}=\omega_{i}^{D}-\frac{\varepsilon }{r_{i}}Y\sin\left(\phi_{i}\right) \end{array}$$

(8) $$\begin{array}{l} {\overset{•}{\omega }}_{i}^{D}=-\varepsilon Y\sin\left(\phi_{i}\right) \end{array}$$

*r*, φ and ω_D_ are the radius, phase and angular frequency variables of a Hopf oscillator respectively (Note: the superscript *D* stands for Decoder module). μ is the parameter that controls the radius of the limit cycle. For μ = 1, it produces a unit circle limit cycle. ε is the coupling factor between the MUX and the Hopf oscillators (Righetti et al., 2006). Because of linearity of the MUX, ε can be computed directly using (9).

(9) $$\begin{array}{l} \varepsilon =P^{+} \end{array}$$

(10) $$\begin{array}{l} P={\left(I-W\right)}^{-1}Q \end{array}$$

*P* is the transformation matrix of the MUX and P^+^ is the pseudo inverse of matrix *P. I* is the identity matrix. *W* and *Q* are the lateral and afferent weight matrices of LAHN respectively. *P* can be derived by virtue of the linearity of LAHN as given in (2).

### DM stage

The purpose of the DM stage is to extract the low-frequency, band limited message signals from the outputs of the FT stage. The DM consists of a layer of Kuramoto oscillators. This shift from Hopf oscillator (in FT stage) to Kuramoto oscillator (in DM stage) is to implement the process of phase synchronization. Kuramoto oscillatory dynamics have been previously implemented to achieve phase synchronization (Kuramoto, 1984). This synchronization in the phase of two oscillators is essential for extracting the message from the FM signal (Haykin et al., 1989) (see Supplementary Material). Each Hopf oscillator in the FT stage is coupled in a one-to-one fashion to a Kuramoto oscillator in the DM stage. The pairs of oscillators (the Kuramoto oscillators of DM and the Hopf oscillators of FT stage) are coupled through their respective phase variables as shown in (11) and (12).

(11) $$\begin{array}{l} {\overset{•}{\gamma }}_{i}=\omega_{i}^{E}+KD_{i} \end{array}$$

(12) $$\begin{array}{l} D_{i}=\sin\left(\phi_{i}-\gamma_{i}\right) \end{array}$$

γ_i_ is the phase variable of *i*th Kuramoto oscillator. It has the same intrinsic frequency, $\omega_{\text{i}}^{\text{E}}$, as that of the encoder oscillators (Equation 1) and *K* is a positive coupling factor (Kuramoto, 1984). This stage is crucial since phase synchronization occurs at this stage and the synchronization dynamics further decodes the low frequency message signal embedded in the output of the Hopf Oscillator (see Supplementary Material).

### LPF stage

*D*_i_ shown in (12) is the output of the decoder which is further passed through a leaky integrator to smoothen the outputs, i.e., low pass filtering (LPF stage). Leaky integrator acts as a low pass filter which further smoothens out the decoded signal, and eliminates any high frequency components present. Dynamics of leaky integrator is given in (13).

(13) $$\begin{array}{l} \frac{d{\overset{\wedge }{s}}_{i}}{dt}=-A\overset{\wedge }{s_{i}}+D_{i}\left(t\right) \end{array}$$

*s*_I_ is the state of *i*th leaky integrator which is the reconstructed version of the input signal *s*_i_(*t*); *A* is the leakage factor which is a positive constant.

Hence the proposed model is a hybrid one consisting of oscillatory layers sandwiching a rate coded layer. Hopf oscillators are used in the model for FM. A layer of linear neurons with lateral connections is used for frequency multiplexing which essentially mixes the FM signals. Hopf oscillators with adaptive frequency are used to track the carrier frequencies of the FM signals. Finally, Kuramoto oscillators are used to demodulate the FM signal and extract the message signal. Parameter values used for the simulation is given in Table 1.

**Table 1:** Parameter values.

**Parameter**	**Value**
μ	1
η_F_	0.01
η_L_	0.01
*dt*	0.01 sec
ϵ	3
*K*	1

## Results

We now test the model described in the previous section on an array of synthetic signals and also on real world EEG signals.

### A. simulation of the model on synthetic signals

The synthetic signals used for the simulation are of the general form *s*(*t*) = *A*_1_sin(ω_1_*t*) + *A*_2_sin(ω_2_*t*).

**Figure 2:**
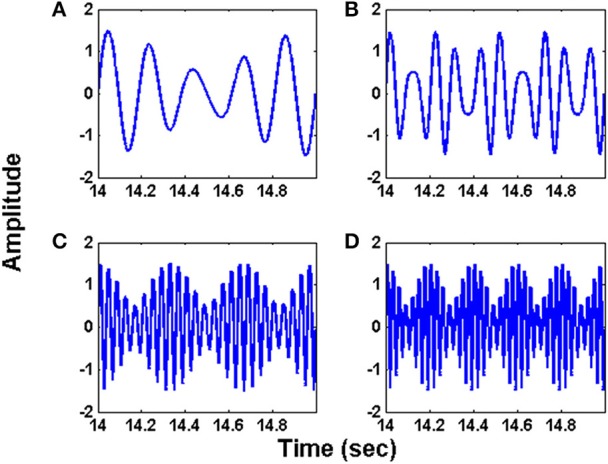
The synthetic input signals used for simulating the oscillatory autoencoder network. The waveforms **(A–D)** follow the equations (14) – (17) respectively.

Specifically, we consider 4 signals shown in (14), (15), (16) and (17) (Figure 2).

(14) $$\begin{array}{l} s_{1}\left(t\right)=\sin\left(10\pi t\right)+0.5\sin\left(12\pi t\right) \end{array}$$

(15) $$\begin{array}{l} s_{2}\left(t\right)=\sin\left(20\pi t\right)+0.5\sin\left(28\pi t\right) \end{array}$$

(16) $$\begin{array}{l} s_{3}\left(t\right)=\sin\left(50\pi t\right)+0.5\sin\left(56\pi t\right) \end{array}$$

(17) $$\begin{array}{l} s_{4}\left(t\right)=\sin\left(70\pi t\right)+0.5\sin\left(80\pi t\right) \end{array}$$

**Figure 3:**
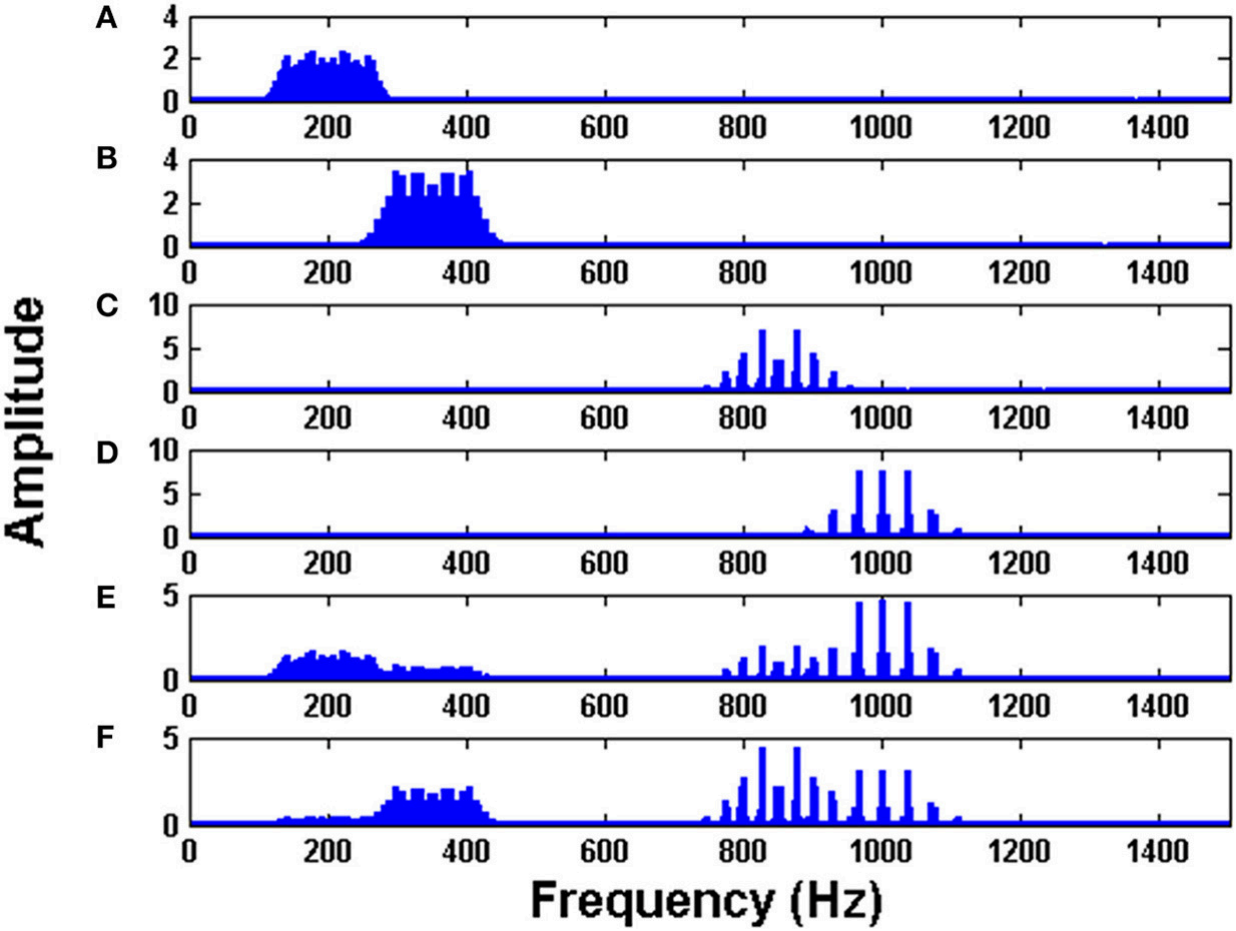
The Fourier transform (FFT) of: **(A)** Modulated signal *O*_1_ **(B)** Modulated signal *O*_2_ **(C)** Modulated signal *O*_3_ **(D)** Modulated signal *O*_4_ **(E)** MUX composite signal *Y*_1_ **(F)** MUX composite signal *Y*_2_. To train the MUX network (LAHN), afferent weights were initialized using random values from uniform distribution [0, 1] and lateral weights were initialized to zero. η_*L*_ and η_*F*_ were taken as 10^−4^.

The initial intrinsic angular frequencies of the FM oscillators are taken as ω^E^ = [200 Hz, 350 Hz, 850 Hz, 1000 Hz]. The input signals, as given by Equations (14)–(17), are used to modulate the encoder oscillators as per Equation (1). Let the resultant frequency modulated signals be *O*_1_, *O*_2_, *O*_3_, *O*_4_ respectively as given by Equation (3). Figures 2A–D shows the waveforms of the input signals (for a short duration) as given by Equations. (14)–(17). Figures 3A–D shows the corresponding frequency spectra. All the frequency spectra are obtained using the Fourier Transformation on the input signals. Figures 3A–D clearly show that the input signals are modulated to the higher frequency regime corresponding to the respective carrier waves.

The modulated signals are passed through a MUX which has two neurons (*n* = 2). The outputs of MUX neurons (MUX composite signal) are *Y*_1_ and *Y*_2_ as per Equation (2). The spectra of *Y*_1_ and *Y*_2_ are depicted in Figures 3E,F. It is evident from Figures 3E,F that the MUX selectively picks and mixes the frequency components of the input signals in such a way that their frequency spectra have minimum overlap. In Figure 3E, one neuron of the MUX was more biased to frequency spectra of *O*_1_ and *O*_4_. In Figure 3F, the second neuron of the MUX was more biased to frequency spectra of *O*_2_ and *O*_3_. The tendency of the hidden layer in a traditional autoencoder to decorrelate the input signals, is manifesting in the present context as a tendency to remix the input signals so that there is minimal overlap in the spectrum (Földiak, 1990).

**Figure 4:**
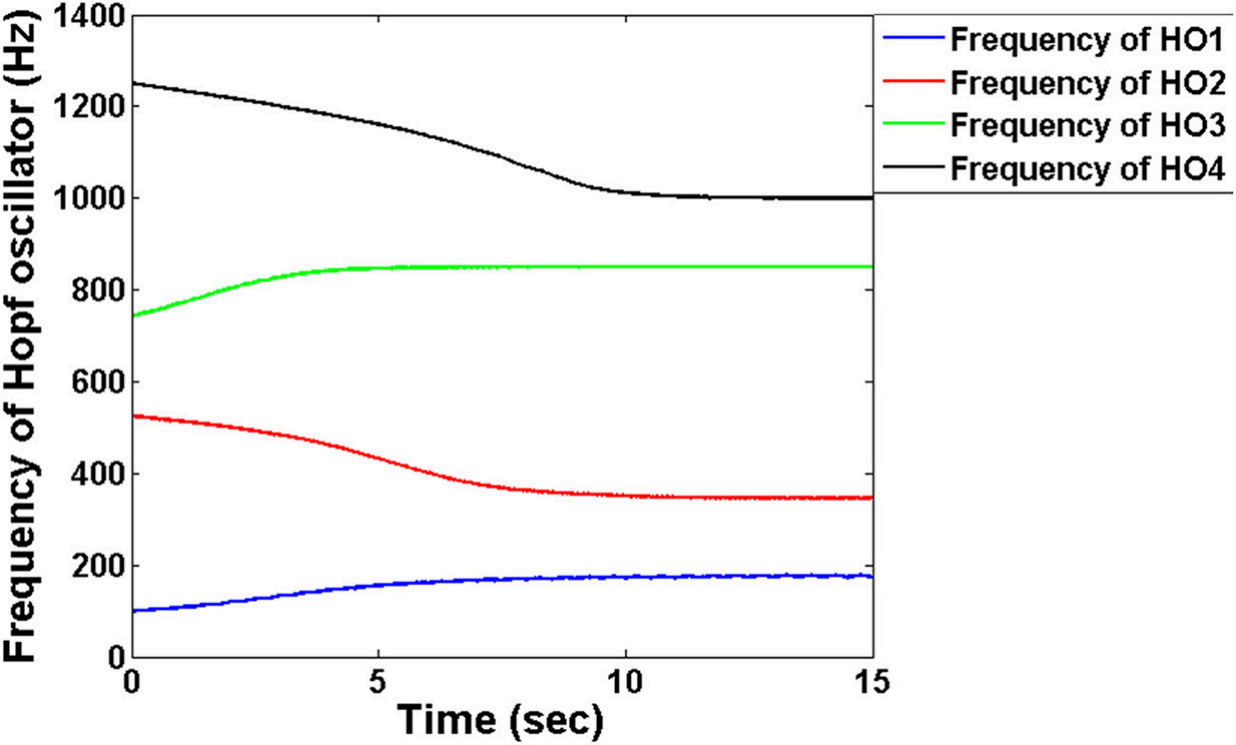
The adaptation of frequencies at the level of the Hopf oscillators in the decoder module: The frequencies are initialized randomly close to the encoder frequencies which via adaptation entrain into each of the four carrier signal frequency. We chose μ = 1 for this simulation.

The FT stage has four Hopf oscillators, which are intended to track the four modulating frequencies. Tracking the frequency is similar to tuning the intrinsic oscillations to that particular channel frequency to fetch the information passed through that respective channel. Figure 4 depicts the frequency adaptation of Hopf oscillators at the FT stage. The intrinsic frequencies of Hopf oscillators are initialized randomly and during the course of time their frequencies get entrained to a specific modulator frequency. Through this adaptation, oscillators are able to select a specific channel of information from a mixture of MUX signals.

**Figure 5:**
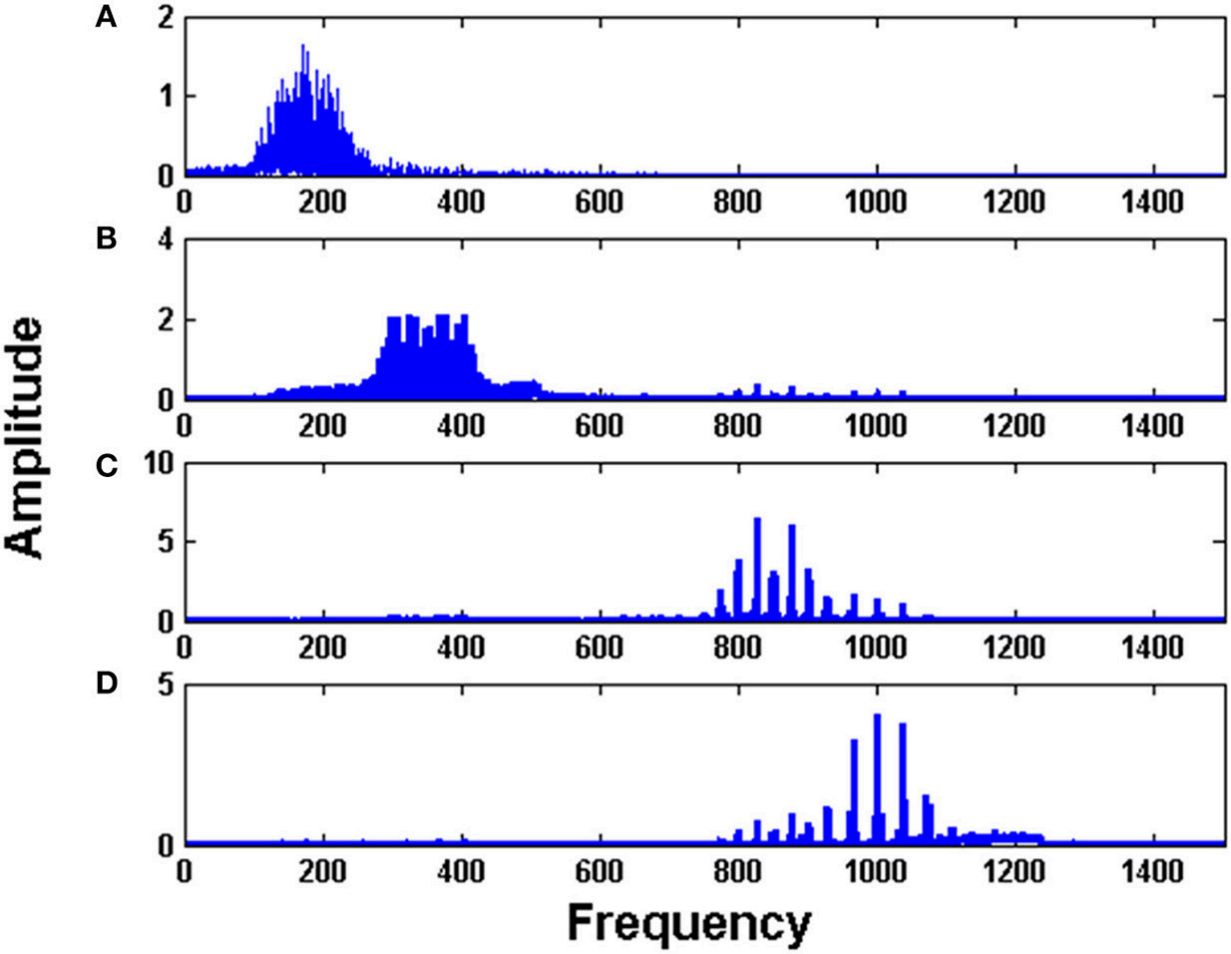
The Fourier spectrum of the four Hopf oscillators: **(A–D)** in the decoder layer show tuning of each oscillator to each channel.

Figure 5 shows the FFT of the four Hopf oscillators' responses. It is evident from the spectrum that each Hopf oscillator is able to pick individual channel that carries the message signal and hence implements the demodulation of the frequency modulated signals. This is an interesting phenomenon which is also observed in the real brain where two cortical regions get entrained to a similar LFP frequency for information transfer or feature binding (Singer and Gray, 1995; Fell and Axmacher, 2011). Synchronization phenomena also circumvent the need for any training between the cortical structures to learn the transmitted information. That is, simply by tuning to a common frequency, two neural structures can communicate over a temporary channel, without any retraining of connections. This is discussed further in detail in the discussion section.

**Figure 6:**
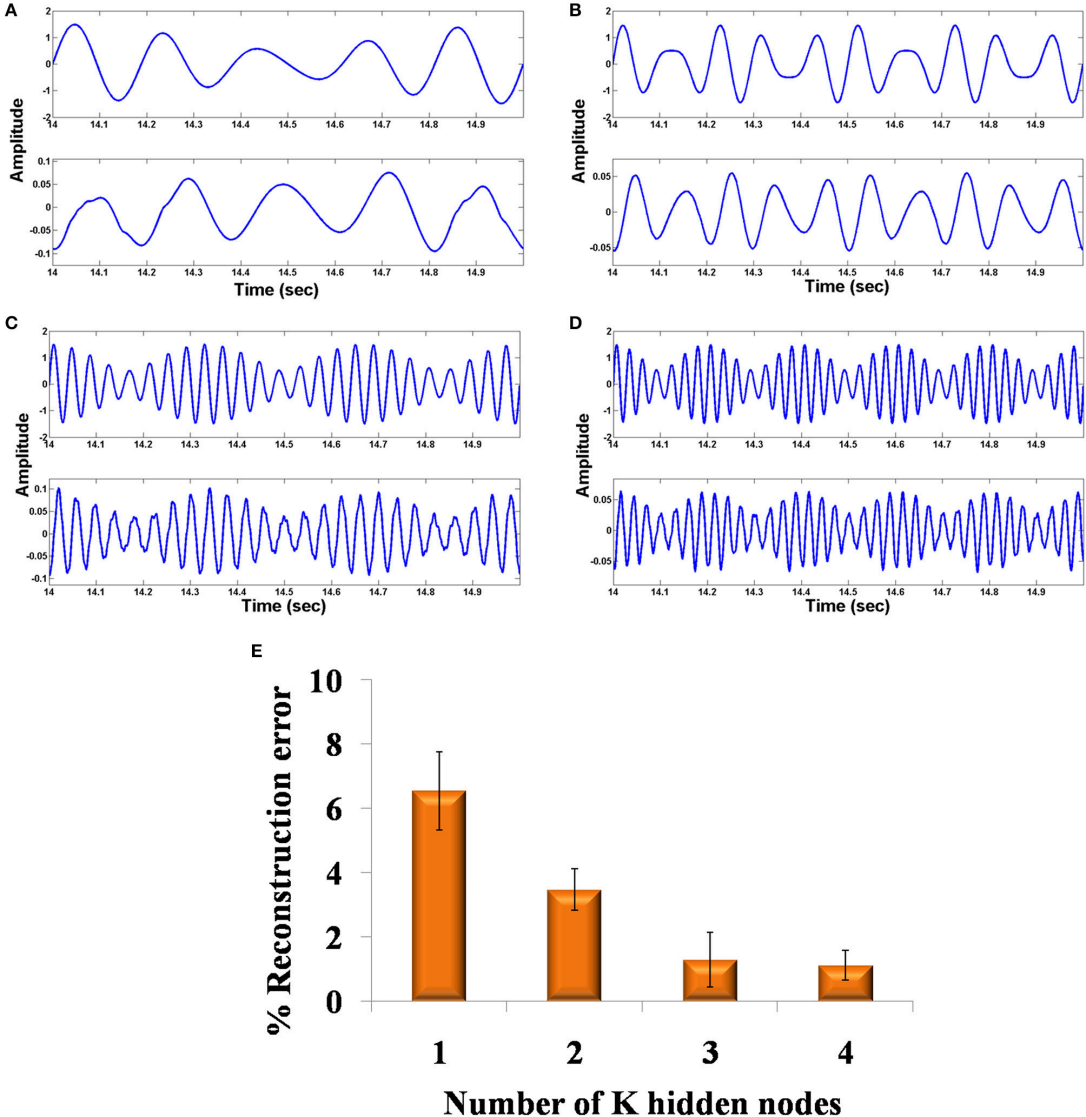
Comparison of original and reconstructed signal from the oscillatory autoencoder: **(A–D)** show the original input (top) to the encoder and the reconstructed signal (bottom) from the decoder using *n* = 2 in the MUX layer. We used *A* = 0.9 for the LPF stage. **(E)** shows the % of reconstruction error with respect to the number of nodes (*n* = 1,2,3,4) in the MUX layer.

Figures 6A–D shows the original and the reconstructed signals (shown for a short duration). The demodulated signals are of lower amplitude and phase shifted compared to the original signal. This can be further corrected using proper amplification and lag shift operation on the output signals. To quantify the accuracy of reconstructed signal, we compute the reconstruction error. This gives an idea on how good the system is with regard to its function as an autoencoder. It is not advisable to directly compare the input and the raw output signal because of the phase shift present in the output signal. To this end, we first corrected the phase shift in the output signal, by computing cross correlation between the input and the output signal. Next, we computed the lag corresponding to the maximum correlation value and circular shifted the output signal using the previously found maximum lag value to correct the phase shift. The percentage (%) reconstruction error is then computed as the deviation of the Pearson correlation coefficient between the input and the phase corrected output signal from unity.

$$\begin{array}{l} \%error=\left[1-corr\left(x,y\right)\right]\times 100 \end{array}$$

where *x* is the input signal and *y* is the amplitude and phase corrected reconstructed output signal. The percentage (%) reconstruction error with respect to the number of nodes in the LAHN network shows a decreasing trend indicating a better recovery of signal with increasing number of nodes in the LAHN layer (Figure 6E). The reconstruction error is computed after phase correction of the output signals as explained above. This result shows that choosing an optimal number of neurons in the hidden layer (based on the reconstruction error), it is possible to form a more efficient abstract representation of the input signal.

### B. simulation of the model on real world signals (EEG signals)

**Figure 7:**
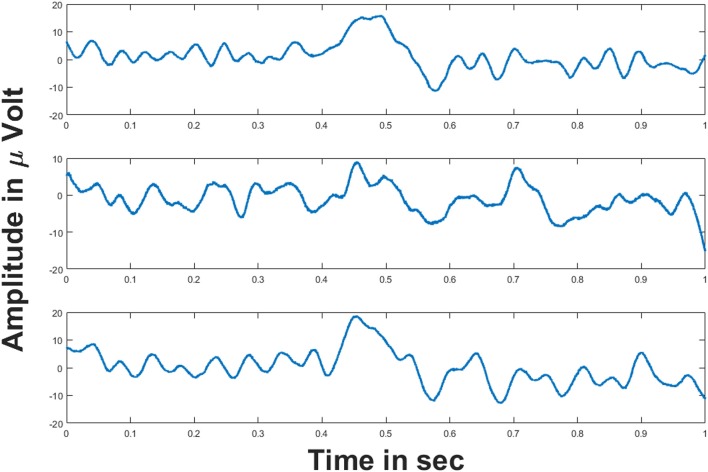
EEG signals from three channels: (Top–Bottom) figures show the EEG signals from C3, Cz and C4 channels respectively for 1 s duration. Voltages on y-axis are given in μ volt.

This section explains the simulation results of the oscillatory autoencoder model on real world data (i.e., data obtained through empirical ways). For this, we considered empirically recorded EEG signals obtained from BCI Competition 2008 - Graz data set B (Leeb et al., 2008). The dataset essentially consists of two class motor imagery EEG signals recorded from three channels (C3, Cz, and C4) (Leeb et al., 2008) at a sampling rate of 250 Hz. Figure 7 shows the 1 s duration EEG signals from the aforementioned channels. These EEG signals were recorded during a motor imagery task. For further information on the experimental protocol the readers may refer (Leeb et al., 2008).

**Figure 8:**
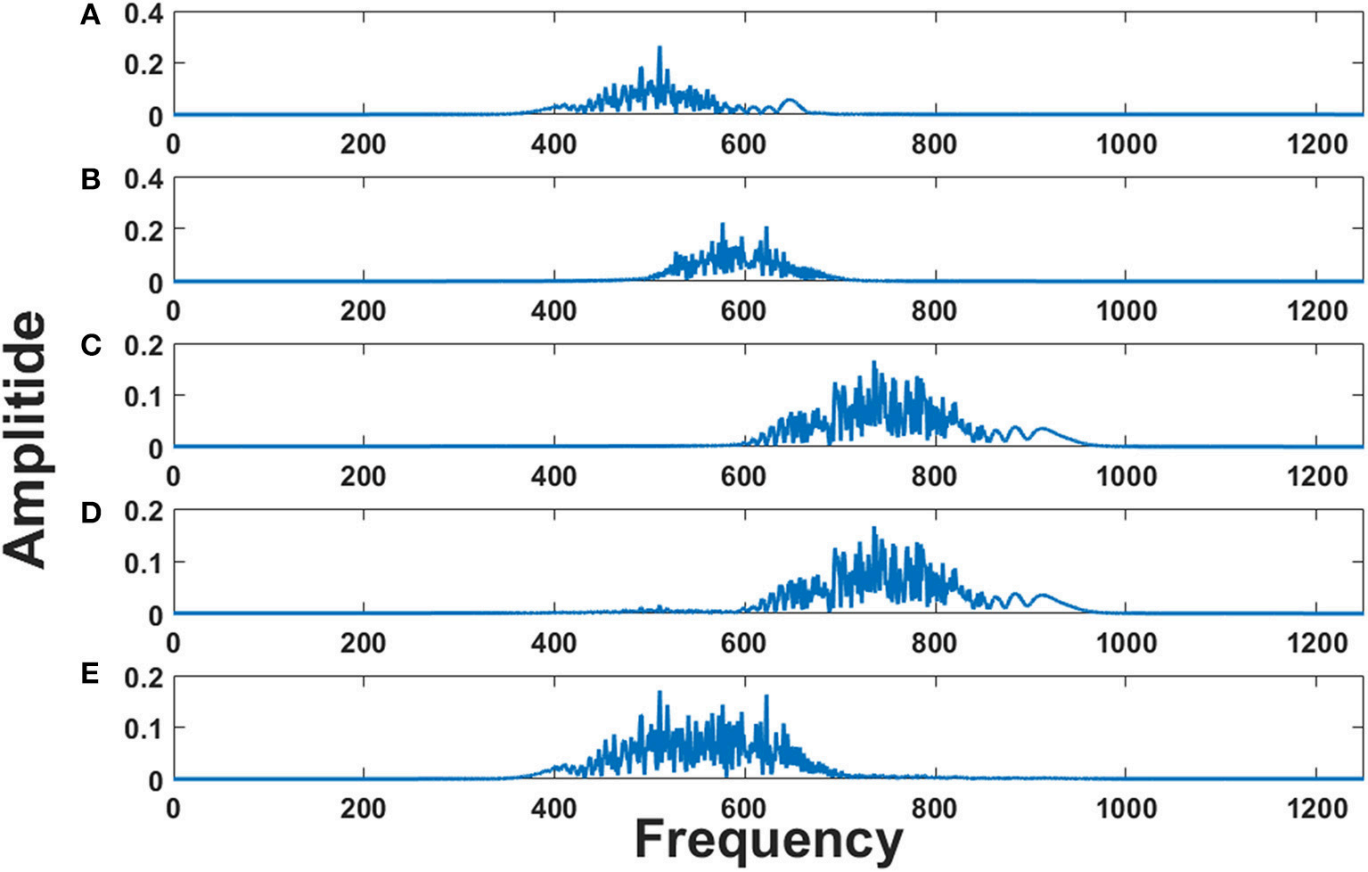
Frequency spectrum of EEG FM signals and EEG MUX signal: **(A–C)** show the frequency spectrum of the EEG frequency modulated signals. **(D,E)** show the frequency spectrum of the composite MUX signal obtained from LAHN. It is vivid from the figures that the MUX signals cover the spectral information of the EEG FM signals and form a low dimensional representation of the same.

These three EEG signals form the input to the model, which is further used to modulate the frequency of the phase oscillators with intrinsic frequencies (500, 600, and 750 Hz). Figures 8A–C shows the frequency spectrum of the frequency modulated signals (EEG FM signals). These signals were further forward passed to the LAHN layer (with two nodes) to get the low dimensional representation of the same and to perform MUX operation. Figures 8D,E shows the frequency spectrum of the MUX-LAHN signals. It is vivid from the figure that each LAHN neuron captures the frequency information of the EEG FM signals and hence forms a low dimensional representation of the raw EEG signals.

**Figure 9:**
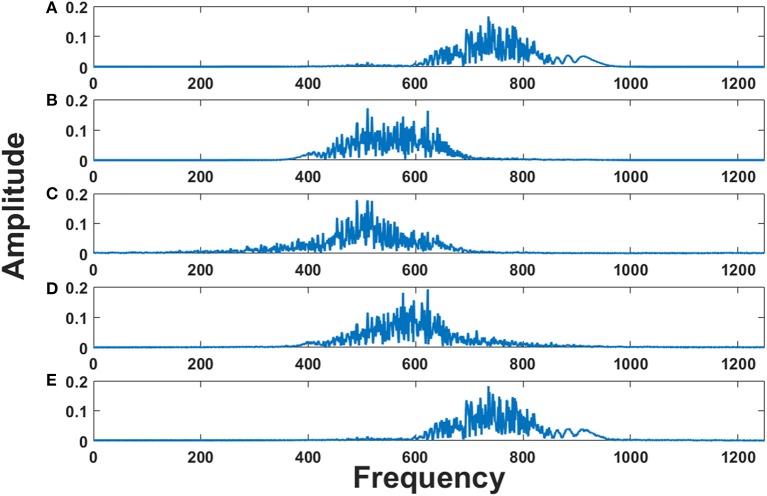
Frequency spectrum of adaptive Hopf oscillators: **(A,B)** show the frequency spectrum of the two MUX signals. **(C–E)** show the frequency spectrum of three adaptive Hopf oscillators and it is evident from the figure that the adaptive Hopf oscillators separate out the frequency spectrum of the EEG FM signals.

Composite MUX signals are further forward passed to the adaptive Hopf oscillators where each Hopf oscillator tunes its intrinsic frequency to each channel frequency. Adaptive Hopf oscillators thus separate the signals from the composite MUX signal (as shown in Figures 9A–B) and this is evident from the frequency spectrum of each Hopf oscillator (Figures 9C–E).

**Figure 10:**
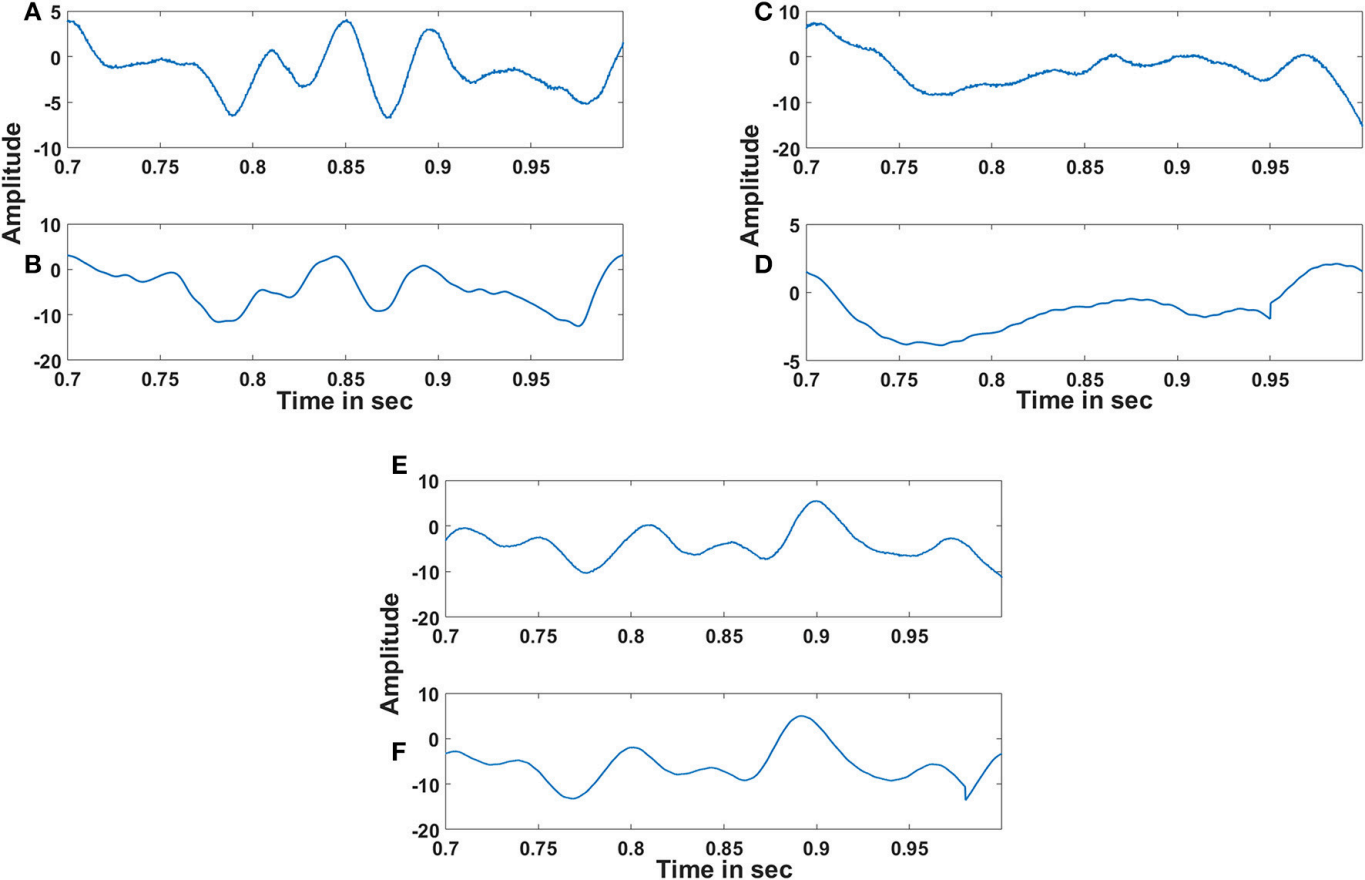
Reconstruction of EEG signals: **(A), (C)** and **(E)** show the original EEG signals recorded from C3, Cz and C4 respectively and **(B), (D)** and **(F)** show the reconstructed EEG signals of the respective channels by the oscillatory autoencoder model. The % of reconstruction error for each reconstruction is 5.8, 6.23, and 3.9% respectively. The absence of noise in the reconstructed signal and also the dimensionality reduction can influence the reconstruction error.

The adaptive Hopf oscillator outputs are further passed to the demodulator Kuramoto oscillators for phase locking and extracts out the embedded EEG signals. Figure 10 shows the reconstructed EEG signal from three channels along with the original signal. The reconstructed EEG signals from the oscillatory autoencoder are smoother than the original EEG signals. This smoothing could be due to the large time scales that govern learning in LAHN and adaptive Hopf oscillatory stage. The subtle changes in the reconstructed signal are due to the lower dimensional representation of the LAHN hidden layer. However, the hidden layer serves as a reliable low dimensional representation of the EEG signals which is further delineated in the following discussion section.

### Comparison of the model result with the benchmark method for dimensionality reduction

In the case of aforementioned EEG result, apart from computing the % reconstruction error, we compare the obtained values with a benchmark dimensionality reduction and reconstruction method to check the goodness of the proposed model. To accomplish this we performed standard Principal Component Analysis (PCA) on the input EEG data to reduce its dimension and further reconstructed back the signal to compute the % reconstruction error. After computing the reconstruction error for each signal, an average reconstruction error is computed to compare it with that of the proposed oscillatory network model. The average reconstruction error of PCA is obtained as 5.68% (computed using MATLAB custom code) considering the first two principal components (because LAHN layer in the model has 2 neurons). The average reconstruction error of the oscillatory autoencoder model is obtained as 5.31%. The average reconstruction error of the proposed oscillatory network model is slightly lower than the standard PCA method by 0.31%. Apart from the decrease in the reconstruction error, the neural attributes of the proposed model and also the theory that the model embodies on the mechanisms of neural information transfer in the brain enhances the significance of the proposed model.

## Discussion

### Summary of the work

We propose here an oscillatory autoencoder that reconstructs the input signal using a well defined encoder and decoder using the principles of FM, MUX, adaptive frequency dynamics, and phase synchronization. We simulated the model using synthetic (linear combination of sinusoids) and also real world EEG signals, thus showing the robustness of the model. The proposed study gives a proof of principle for the potentiality of the oscillatory neural networks in non-trivial applications where oscillations are seldom used such as autoencoder (problem addressed in this paper), feature extraction, clustering, classification etc where mostly rate coded networks are used. The criticality of oscillations in neurobiology, as mentioned below, is the motivation of this work.

### Criticality of the oscillations

Although in computational neuroscience literature, oscillatory neurons are not as common as rate-coded or spiking neuron models, oscillations figure prominently in experimental neurobiology. There exists a large corpus of experimental literature that correlates animal behavior with the aspects of neural oscillations (Buzsáki, 2002; Lisman and Buzsáki, 2008; Adhikari et al., 2010; Fell and Axmacher, 2011). Instances can be found from experimental neurobiology wherein all the major components of neural information processing viz., communication, representation and learning are implemented by neural oscillations. Colgin et al. (2009) reported that CA1 region of hippocampus communicates with Medial Entorhinal Cortex (MEC) via fast gamma synchronization (65–140 Hz) and with CA3 region via slow gamma synchronization (25–50 Hz) (Colgin et al., 2009). That is, by changing the frequency of the signal, it is possible to select the route by which communication takes place. Spatially distributed neurons can encode for several individual features of an object by synchronizing the neural discharges of the features, a phenomenon known as feature binding (Singer and Gray, 1995). For instance, the presentation of an optimally oriented bar gives rise to synchronized spiking of neurons, which are spatially distributed, in the area 17 of the visual cortex (Gray and Singer, 1987). Synchronization in the neural discharge is mirrored in the phase of the corresponding oscillatory LFP activity too. Hence there is a high correlation between the spike timing with the phase of the LFP oscillations. In case of feature binding, synchronization may not sometimes be evident from the spiking activity of the neurons, but the LFP activity shows robust phase synchronization (Alonso and Garcia-Austt, 1987; Buzsáki et al., 1992). Thus understanding the system dynamics in terms of oscillations becomes crucial. In the perspective of learning, a volley of high frequency pre-synaptic pulses with simultaneous depolarization at the postsynaptic side leads to Long Term Potentiation (LTP) (Bliss and Lømo, 1973; Lüscher and Malenka, 2012). These high frequency spikes can be correlated with the corresponding LFP oscillations. Hence the same LTP defined in terms of the spikes can be redefined using oscillatory LFP (Chauvette et al., 2012). Oscillations also have a pivotal role in cognition in both normal and pathological conditions. For example, the disconnectivity hypothesis of schizophrenia relates the disease symptoms to the dysfunction in the communication between different brain regions (Williams and Boksa, 2010). Gamma rhythm has been reported to have a role in the information transfer between the brain regions (Gray et al., 1989). In the early onset schizophrenic patients there is a reduction in the power of the gamma oscillation in the Prefrontal Cortex (PFC) a reason accounted for impaired working memory (Haenschel et al., 2009). Longer time scale oscillations like circadian rhythms are also known to play a critical role in major psychological disorders like bipolar disorder, depression, addiction (McClung, 2007; Alloy et al., 2015). Thus, from circadian to high gamma rhythms, oscillator models can be used to describe brain dynamics over a wide range of frequencies.

### Relation of neural information transfer to radio communication principles

The proposed work reinforces the hypothesis of information transfer between the brain regions to FM radio principles proposed by Hoppensteadt and Izhikevich (1998), that cortical areas communicate each other by making sure that their oscillations satisfy a resonant condition (Hoppensteadt and Izhikevich, 1998). They hypothesized that cortical oscillations are frequency modulated (FM) and, when the frequencies of two cortical areas match, they communicate by phase modulation. Thus, cortical communication is proposed to operate on the lines similar to FM radio. Although their paper proposed that signals can be frequency modulated and demodulated, it does not present how these concepts could actually be exploited to perform autoencoding, i.e., the input messages getting frequency modulated, multiplexed, demultiplexed and frequency demodulated. The proposed oscillatory autoencoder model realizes this concept by invoking the adaptive frequency and phase synchronization dynamics which take care of the frequency tuning to the incoming FM signal and hence offers a neurally plausible mechanism for the signal transmission and reconstruction (autoencoding) in the brain. This is achieved by the use of Hopf and Kuramoto dynamics. Both Kuramoto and Hopf oscillators have been previously used as models of neural oscillations in many instances (Cumin and Unsworth, 2007; Righetti et al., 2009). The Kuramoto model has been used to explain neuronal synchronization in large connected networks (Cumin and Unsworth, 2007) and especially building generative models of cortical oscillations (Breakspear et al., 2010). On the other hand, adaptive Hopf oscillators have been used for the generation of rhythmic output patterns such as central pattern generators involved in locomotion (Ijspeert et al., 2005). However, we have not come across any literature exploiting the phase synchronization properties of Kuramoto oscillators and adaptive frequency aspects of Hopf oscillator to model frequency multiplexing and demultiplexing. One of the interesting achievements of the proposed model is to show that Kuramoto—Hopf oscillator combination could act as a neural phase locked loop (PLL) which can be used to decode information from a given cortical region.

### Possible applications of the model

Autoencoder networks are usually constructed out of rate coded neurons, though in the recent times autoencoder networks with spiking neurons have also been proposed (Burbank, 2015). In its simplest form, a rate-coded autoencoder is a feedforward network with a single hidden layer and is trained such that the target output is the same as the input; the hidden layer has fewer neurons than the input or the output layer. Then the hidden layer learns to represent the input using fewer dimensions and therefore achieves dimensionality reduction of the input space (Hinton and Salakhutdinov, 2006). A similar reduction is achieved in the proposed oscillatory model since the hidden layer, LAHN, is of lower dimension than the input layer. The connection between Hebbian learning rule and Principal Component Analysis (PCA) is not a new idea since Oja has previously shown how a linear neuron adapting its synaptic weight connections using Hebbian learning rule can converge to the first principal component of the input data (Oja, 1989). This was further extended by Sanger using an asymmetric Generalized Hebbian Algorithm (GHA) learning rule that makes the network to learn the first n principal components (Sanger, 1989) instead of just one principal component. Hebbian/anti-Hebbian network also comes under the category of subspace learning network. This type of network, reduces the input data dimension by learning the principal subspace of the input data (Földiak, 1990; Hu et al., 2015; Pehlevan et al., 2015). Other neural networks in this line are subspace network, Rubner's network (Rubner and Tavan, 1989; Rubner and Schulten, 1990) etc. Although these networks were initially modeled to explain the computations behind the processing of streaming sensory inputs, the synaptic plasticity rules based on the local activity of the neurons neuronal activity were postulated rather than derived from a cost function (Földiak, 1990). This gap was further bridged by computing the local learning rules from a principled cost function (Hu et al., 2015; Pehlevan et al., 2015). Changing the non-linearity of the neuronal activation function explained the potentiality of these networks in extracting the higher order moments of the input data and hence qualified them as the neural architectures for Independent Component Analysis (ICA) (Oja, 1997). The aforementioned studies prove the criticality of this type of network in various applications that include subspace learning, source separation problem, dimensionality reduction etc.

This dimensionality reduction has further implications especially in EEG processing. The model reconstructs the original EEG signals from their lower dimensional LAHN representations. This means that these LAHN signals can serve as the reliable representations especially for high channel EEG signals. These representations could potentially be useful in BCI related processing such as classification of EEG signals, feature clustering, movement signature detection etc. The EEG signals used for the model simulation are two class motor imagery signals which are of particular interest in BCI application. Hence the proposed model not only provides a biologically plausible explanation for the information transfer in the brain but also shows its possible potential application in BCI related EEG processing. Another important feature that makes the current model suitable for EEG processing is its ability to average out the noise present in the input signal. As shown in the results section, the input EEG signals have high frequency ripples in its original form which is further averaged out to produce a smooth reconstructed signal as the output from the model (Figure 10). This could possibly be due to the large temporal scale Hebbian learning that happens in the hidden LAHN layer which could thus average out the noise present in the input.

### Future extensions of the proposed model

A possible extension of the current model could be to add additional circuitry that will enable routing of the signal from the *i*th input channel to the *j*th output channel. It must be possible to choose the input/output channels to be coupled through another layer that projects to the current LAHN layer that performs multiplexing of FM signals. In such an extended model, the LAHN layer and the additional circuitry for route selection can be compared to the functions of the thalamus with respect to cortico-thalamic information processing. Hence the proposed model serves as the proof of principle for the potentiality of the oscillatory neural networks in information transfer i.e., encoding and decoding of real world signals using the principles of modulation, MUX, frequency adaptation and phase synchronization and also shows its possible potential role in EEG related applications. Another direction that the proposed model could possibly take is to pick the brain components from the EEG signals. By *brain component* we mean the sources inside the brain responsible for the generation of the EEG signal. Current approaches like Independent Component Analysis (ICA) require manual selection of components which has a source inside the brain for further analysis. We envisage that using a hierarchical network of LAHN, we could possibly isolate the brain components better due to its inherent ability to filter out noise. As a future work, we envisage that the current model could be possibly used (may be by invoking minor changes) to study the EEG related phenomena like mu band Event Related Desynchronization (ERD), Visual Evoked Event Related Potential (ERP) etc which can possibly shed light on the neural principles behind the occurrence of these phenomena.

## Conclusion

We propose a hybrid oscillatory network model that performs the function of an autoencoder. Using this network, we are able to encode the information onto oscillations, reduce the dimensionality of information and effectively decode them using a neural phase locked loop. The model was successfully applied to both synthetic as well as real world EEG signals. Hence the proposed model shows an oscillatory neural framework in describing information transfer in the brain. By reconstructing the EEG signals from its abstract representations in the hidden layer we have shown the model's ability in better feature extraction of EEG signal which is a critical part in EEG processing. Finally, we conclude that exploring the universality of oscillator networks would open avenues for developing an entirely new class of neural network models that describe brain function in terms of oscillatory properties— amplitude, frequency, and phase. The whole motivation of this work was to show a proof of principle for the potentiality of the oscillatory networks in other domains where usually rate coded or spiking neurons were used. In the future, we plan to apply the model to a wider variety of real world time series signals.

## Data availability statement

All the simulations are done in MATLAB R2016a and the code is made available in Model DB repository. URL is http://senselab.med.yale.edu/ModelDB/showModel.cshtml?model=243595

## Author contributions

All authors contributed equally to the work. KS performed designing, coding, analysis of the model, and manuscript preparation. VM performed analysis of the model, and manuscript preparation. VC performed designing the model, analysis of the model and manuscript preparation.

### Conflict of interest statement

The authors declare that the research was conducted in the absence of any commercial or financial relationships that could be construed as a potential conflict of interest.
