# Supplementary material for: An Oscillatory Neural Autoencoder Based on Frequency Modulation and Multiplexing
Source: Front Comput Neurosci. 2018 Jul 10;12:52. doi: 10.3389/fncom.2018.00052 (PMC6048285; doi:10.3389/fncom.2018.00052)
Supplement: Supplementary file 1 [file Data_Sheet_1.DOCX]

Supplementary Material

An Oscillatory Neural Autoencoder based on Frequency Modulation and Multiplexing

Karthik Soman, Vignesh Muralidharan, V. Srinivasa Chakravarthy*

*** Correspondence:** Dr. V. Srinivasa Chakravarthy: schakra@iitm.ac.in

# Dynamics of Demodulation using Kuramoto oscillator

Let phase dynamics of the message carrying modulated signal be:

$\mathop{\theta_{1}}\limits^{\bullet} =\omega+m(t)$ (A.1)

Let the oscillator with phase variable *θ_2_* be coupled to *θ_1_* as (A.2)

$\mathop{\theta_{2}}\limits^{\bullet} =\omega+\sin(\theta_{1}-\theta_{2})$ (A.2)

*m(t)* is the message signal and *ω* is the angular frequency of the oscillators.

Let *ψ* represents the phase difference between the oscillators.

$\psi=\theta_{1}-\theta_{2}$ (A.3)

Hence

$\psi^{\bullet} =\mathop{\theta_{1}}\limits^{\bullet} -\mathop{\theta_{2}}\limits^{\bullet}$ (A.4)

$$=m(t)-\sin(\theta_{1}-\theta_{2})$$

$\psi^{\bullet} =m(t)-\sin(\psi)$ (A.5)

At steady state

$$\psi^{\bullet} =0$$

Hence from (A.5)

$$m(t)=\sin(\psi)$$

When the oscillators tend to phase lock (Kuramoto oscillators tend to synchronize their phases)

$$\psi\to0$$

Hence $\sin(\psi)\to\psi$ (Small angle approximation)
